# A report card assessment of the prevalence of healthy eating among preschool-aged children: a cross-cultural study across Australia, Hong Kong, Singapore, and the US

**DOI:** 10.3389/fnut.2024.1428852

**Published:** 2024-08-21

**Authors:** Alison Wing Lam Wan, Kevin Kien Hoa Chung, Jian-Bin Li, Shebe Siwei Xu, Derwin King Chung Chan

**Affiliations:** Department of Early Childhood Education, The Education University of Hong Kong, Hong Kong, Hong Kong SAR, China

**Keywords:** healthy eating, prevalence, report card, eating behaviours, family home food environment, preschool-aged children, obesity

## Abstract

**Objective:**

This study aimed to initially adopt an International Healthy Eating Report Card for Preschool-Aged Children to assess the prevalence of healthy eating behaviours and favourable family home food environments (FHFEs) among preschool-aged children in Australia, Hong Kong, Singapore, and the US. We also examined which cultural contexts would exhibit significant differences in the report card scores among the four cultural contexts.

**Methods:**

In this cross-cultural study, 2059 parent–child dyads, with approximately 500 dyads in each cultural context, were recruited. The parents were asked to complete the validated International Healthy Eating Report Card Scale to assess the dimensions of the Report Card [i.e., Indicator of Children’s Eating Behaviours: (1) Children’s Dietary Patterns and (2) Children’s Mealtime Behaviours, and Indicator of FHFEs: (3) Parental Food Choices and Preparation, (4) Home Healthier Food Availability and Accessibility and (5) Family Mealtime Environments]. Each indicator received a letter grade [i.e., A (≥80%) = excellent, B (60–79%) = good, C (40–59%) = fair, D (20–39%) = poor, F (<20%) = very poor and including the plus (+) and minus (−) signs] to represent the proportion of participants who could meet the predefined benchmarks. We also employed ANCOVA and Bonferroni’s post-hoc test to examine the differences in the report card scores between the four cultural contexts. A significance level was set at *p* < 0.05.

**Results:**

The average overall report card grade across the four cultural contexts was “B−” (Good), ranging from “C+” (Singapore and the US) to “B−” (Australia and Hong Kong). The average grade for Children’s Eating Behaviours was classified as Fair (“C−”), while the average grade for FHFEs was classified as Good (“B+”) for all cultural contexts. A comparison of the overall report card scores revealed that Australia exhibited a significantly higher report card score than Singapore and the US, while Hong Kong achieved a significantly higher score than Singapore.

**Conclusion:**

The International Healthy Eating Report Card provided an overview of the prevalence of healthy eating in different cultural contexts. We believe that the International Healthy Eating Report Card may offer new perspectives on interventions for fostering healthy eating in young children.

## Introduction

Overweight and obesity are highly prevalent health conditions. In 2022, more than 1 billion people worldwide, including 37 million children aged less than 5 years old, are considered to be overweight or obese (1). This alarming prevalence of obesity reveals that childhood obesity has become a severe modern-day epidemic (2). The effective reduction and prevention of overweight and obesity strongly depend on individuals adopting healthy eating behaviours, such as increasing the consumption of a variety of nutrient-dense foods while replacing energy-dense options (3). Promoting healthy eating practices among young children is imperative, as early adoption of healthy eating habits may have a lasting impact on an individual’s dietary behaviours, potentially reducing the risk of developing obesity-related health complications during adulthood (4, 5).

However, there is a recent trend towards unhealthy dietary patterns among young children across the globe, namely frequent consumption of energy-dense nutrient-poor snack foods, insufficient consumption of fruits and vegetables, low dietary diversity, and irregular breakfast consumption (6–8). For instance, in the United States (US), fewer than 13% of children aged 1–8 years met the daily vegetable intake recommendation, while 95.2% of them exceeded the recommended daily limit for solid fats and added sugar (9). Similarly, almost 95% of Australian children aged 2–8 years did not meet the recommended daily intake of vegetables, and approximately 50% of them consumed unhealthy snacks every day (10). Similar trends are observed in Asian societies. In Singapore, a study by Lim and Teoh (11) indicated that, on average, 84% of young Singaporean children aged 2–6 years could not meet the recommended servings of dairy products, fruits and vegetables. A cross-sectional survey revealed that young Hong Kong children aged between 2 and 4 years experienced prolonged dependence on formula milk (87.7%) and excessive consumption of unhealthy snacks and sugar-sweetened drinks (90%) in their diet, which led to a reduction in their consumption of grains, vegetables and fruits (12).

These findings underscore a critical need for promoting healthy eating habits among young children. One step towards achieving this goal is to conduct an in-depth evaluation of the current prevalence of healthy eating among young children. Such evaluations play a pivotal role in identifying prevalent eating problems and raising public awareness, ultimately finding possible solutions and fostering healthy eating promotion in public health initiatives. In addition, children’s dietary patterns and even mealtime behaviours are significantly influenced by family home food environments (FHFEs), including parents’ behavioural factors (i.e., parental food choices and preparation) and both physical and social home environment factors (i.e., home food availability and accessibility and family mealtime environments) (13–18). Therefore, it is crucial to examine these influencing factors to understand the extent to which children could get involved in favourable FHFEs which, in turn, promotes particular aspects of FHFEs to cultivate children’s healthy eating habits.

Despite the critical role of healthy eating behaviours and FHFEs in preventing childhood obesity, there is a lack of a systematic assessment framework that consistently examines the extent to which children can adhere to healthy eating behaviours and engage in favourable FHFEs within a particular region/country. Additionally, studies providing a comprehensive overview of healthy eating specifically in preschool-aged children are lacking as existing national and research surveys on healthy eating may not target this specific age group (10, 19), and some aspects of the eating behaviours of preschool-aged children, such as dietary variety, the use of formula milk, mealtime behaviours, and even the assessment of FHFEs may not be comprehensively covered (20, 21). These gaps highlight the need for developing a robust assessment framework [e.g., a report card approach (22)] to advance the understanding of the prevalence of healthy eating among preschool-aged children.

### Application of the report card framework

A report card approach may offer a comprehensive and succinct method to evaluate the prevalence of a particular health behaviour within a specific region or country. This approach was initially adopted for healthy eating behaviours to provide a holistic and precise overview of how well preschool children in Hong Kong could adhere to the standards and recommendations of the Department of Health of the Hong Kong Special Administrative Region regarding healthy eating behaviours and be involved in favourable FHFEs by displaying letter grades (i.e., A+ to F) (23). However, this region-specific report card may only be applicable in the context of Hong Kong. Therefore, in recognition of the limitations of this regional report card, a recent study developed an international version of the Healthy Eating Report Card by incorporating the standards and recommendations of global health authorities, such as the World Health Organisation and the Centers for Disease Control and Prevention, and some relevant research evidence of healthy eating (24). This international version of the Healthy Eating Report Card has been validated in Australia, Hong Kong, Singapore, and the US with robust psychometric properties (24).

In addition to assessing the prevalence of healthy eating behaviours and favourable FHFEs among preschool-aged children within countries/regions, this international report card of healthy eating allows cross-cultural comparisons to provide insights and elucidate the diversity of eating behaviours and FHFEs among different countries/regions. Displaying the letter grades and calculating report card scores can help identify countries/regions that successfully promote healthy eating among young children while highlighting specific areas with lower grades for improvement. This approach not only enhances public awareness but also stimulates broader public discussion and ultimately informs public health initiatives and policy changes related to healthy eating (25). Although the International Healthy Eating Report Card has been developed, it has yet to be applied across various countries or regions to effectively assess and compare the prevalence of healthy eating.

### Present study

To address the knowledge gaps in comprehensively evaluating the prevalence of both healthy eating behaviours and favourable FHFEs within different regions/ countries, the present study, therefore, aims to:

(1) Utilise the International Healthy Eating Report Card to cross-culturally examine the patterns of healthy eating among preschool-aged children in four cultural contexts, including Australia, Hong Kong, Singapore and the US.
(2) Examine which cultural contexts would exhibit significant differences in the scores of children’s eating behaviours, FHFEs and overall report card (combined scores of all the indicators) among the cultural contexts.

Building on the frameworks established by prior studies on the Healthy Eating Report Card (23, 24), we will use the five indicators of the report card for assessing children’s eating behaviours [(1) Children’s Dietary Patterns and (2) Children’s Mealtime Behaviours], and FHFEs [(3) Parental Food Choices and Preparation, (4) Home Healthier Food Availability and Accessibility, and (5) Family Mealtime Environments] within the four cultural contexts. The evidence-based benchmark will subsequently be developed to guide the process of assigning letter grades to each indicator reflecting the proportion of children who meet the recommendations and guidelines of the global health authorities for healthy eating behaviours and get involved in favourable FHFEs. We believe that the findings of this study could help us gain a better understanding of the prevalence of healthy eating across developed Asian and Western societies.

## Method

### Participants

The current study utilised the dataset from a previous study that focused only on the validation of the International Healthy Eating Report Card Scale (24), and descriptive analyses of the patterns of healthy eating were not conducted. The dataset was collected through a Qualtrics online survey panel in early December 2022, which included 2059 participants (2059 parent–child dyads) recruited from Australia (*n* = 500), Hong Kong (*n* = 552), Singapore (*n* = 507), and the US (*n* = 500). We selected these four cultural contexts due to their representation in developed countries/regions (Human Development Index ≥0.9) and the inclusion of both Asian and Western cultures, ensuring a comprehensive comparative analysis of children’s eating behaviours and FHFEs across different cultural settings. The participants met the following inclusion criteria: (i) participants reside in Australia, Hong Kong, Singapore, or the US, (ii) parents have at least one child, (iii) the age of parents’ oldest child ranges from 2 to 6 years old and (iv) their child does not have any medical conditions. The study population included mothers (60.95%), followed by fathers (37.49%) and other guardians (1.56%). The average age of the parent participants was 36 years (*SD* = 6.99), and they had an average of 1.77 children (*SD* = 0.94). There were 93.82 and 72.41% of working fathers and mothers, respectively, and 57.67% reached or exceeded the median household income in their respective cultural contexts. The children had a mean age of 4.56 years (*SD* = 1.32), with 49.9% being boys and 82.61% living in two-parent families.

To assess the representativeness of the sample data with respect to the general population, the sociodemographic characteristics of the sample were compared with recent data from the population censuses of the four cultural contexts (i.e., Australia, Hong Kong, Singapore and the US). Sociodemographic differences between the samples and the population censuses in the four cultural contexts were small (Cohen’s *w* < 0.3 and Cohen’s *d* < 0.5). The sociodemographic characteristics for each cultural context and the effect sizes for the comparisons between the samples and the population census data are shown in Table 1.

**Table 1:** Sociodemographic characteristics for each cultural context and the effect sizes for the comparisons between the samples and the census of population data.

Sociodemographic	Census	Sample	Effect size
**Average number of children in household**			
Australia	1.9	2.03 (*SD* = 0.96)	0.14
Hong Kong	1.4	1.30 (*SD* = 0.54)	0.19
Singapore	1.3	1.70 (*SD* = 0.82)	0.48
The United States	1.9	2.11 (*SD* = 1.14)	0.15
**Percentage of working fathers (%)**			
Australia	91.9	91.0	0.01
Hong Kong	90.7	98.7	0.12
Singapore	89.6	96.6	0.10
The United States	94.4	87.8	0.14
**Percentage of working mothers (%)**			
Australia	75.0	60.5	0.15
Hong Kong	74.5	76.3	0.02
Singapore	76.2	85.2	0.09
The United States	67.9	66.9	0.01
**Percentage of children living in a single-parent family (%)**			
Australia	14.2	21.1	0.09
Hong Kong	6.0	4.1	0.03
Singapore	8.58	3.3	0.08
The United States	29.8	42.4	0.12
**Percentage of families above median household income (%)**			
Australia	53.4	51.2	0.02
Hong Kong	44.3	37.7	0.06
Singapore	60.5	87.2	0.23
The United States	49.8	50.0	<0.01

The census data are sourced from the Australian Bureau of Statistics (Australia), the Census and Statistics Department (Hong Kong Special Administrative Region), the Department of Statistics (Singapore), and the U.S. Census Bureau (United States). Sociodemographic differences between the samples and the census data in the four cultural settings were small (Cohen’s *w* < 0.3 and Cohen’s *d* < 0.5).

### Procedure

The prior recruitment protocol was approved by the Human Research Ethics Committee at the institution of the primary author (Reference number: 2022-2023-0033). This cross-cultural comparative study aimed to adopt a report card approach to reveal the prevalence of healthy eating among children in four cultural contexts and to examine the significant differences in the scores of the report card across these cultural contexts. After providing informed consent, the parent participants were asked to complete an online self-report questionnaire once regarding their children’s eating behaviours, parental food choices and preparation practices and home food environments.

### Measures

The International Healthy Eating Report Card Scale (IHERCS) (24) was adopted to assess children’s eating behaviours and FHFEs. This scale was cross-culturally validated in a previous study. It includes 43 items aligning with the dimensions of the International Healthy Eating Report Card [i.e., Indicator of Children’s Eating Behaviours: (1) Children’s Dietary Patterns and (2) Children’s Mealtime Behaviours], and Indicator of FHFEs: [(3) Parental Food Choices and Preparation, (4) Home Healthier Food Availability and Accessibility and (5) Family Mealtime Environments]. The participants responded to the items on a 5-point Likert scale [i.e., ranging from 1 (*never*) to 5 (*always*)], an open-ended question and multiple-choice questions. Additionally, participants responded to demographic questions, including their parent’s age, gender, number of children they had, employment status, education attainment and monthly household income. The participants also provided their child’s date of birth and gender. The assessment tool of the IHERCS is available in the supplementary document of the previous study (24).

### Analysis

We followed an analysis protocol identical to that used in an initial study regarding the Healthy Eating Report Card to determine the letter grade for each indicator (23). A novel benchmark framework that aligned with the components of the International Healthy Eating Report Card was established based on the recommendations and guidelines from global health authorities (26–33) and the relevant literature (17, 34–40). This framework was designed to determine the letter grade for the indicators based on participants’ responses to the IHERCS, representing the percentage of participants who met the predefined benchmarks. A sub-grade was also assigned to each item of the indicators. The response criteria for meeting benchmarks are displayed in Appendix A. A grading rubric from a previous study was also adopted to assess how well children adhered to guidelines for healthy eating behaviours and got involved in favourable FHFEs [i.e., A (≥80%) = Excellent, B (60–79%) = Good, C (40–59%) = Fair, D (20–39%) = Poor, F (<20%) = Very Poor with plus (+) and minus (−) signs to indicate the upper or lower 5% of the grade range] (23). The benchmarks and the grading system are shown in Tables 2, 3, respectively.

**Table 2:** Indicators and benchmarks in International Healthy Eating Report Card for Preschool-Aged Children.

Indicator	Benchmark
**Indicators of children’s eating behaviours**	
(1) Children’s dietary patterns	% of children who eat breakfast daily (27)
	% of children who do not have a formula milk-drinking habit (40)
	% of children who eat a variety of foods from each of the five main food groups^*^ daily (28)
	% of children who have an adequate fluid intake daily (29) (i.e., 1,300 mL/day for children aged 2–3; 1,600 mL/day for children aged 4–8)
	% of children who have an adequate vegetable intake daily (33) (i.e., 1 cup of raw or cooked vegetables per day for children aged 2–3; 1.5 cups of vegetables per day for children aged 4–8)
	% of children who have an adequate fruit intake daily (33) (i.e., 1 cup of fruit per day for children aged 2–3; 1.5 cups of fruit per day for children aged 4–8)
	% of children who eat healthy snacks between meals (30)
	% of children who consume unhealthy snacks fewer than three times per week (26, 34, 36)
	% of children who drink sugar-sweetened beverages fewer than three times per week (26, 34, 36)
(2) Children’s mealtime behaviours	% of children who are not picky eaters (35)
	% of children who are not slow eaters (35)
	% of children who remain seated at the table for most of the meal (35)
	% of children who do not refuse to eat at meals (35)
	% of children who do not require parental feeding assistance to finish most meals (35)
	% of children who eat an adequately sized meal (35)
	% of children who do not throw a tantrum during mealtimes (35)
**Indicators of family home food environments**	
(3) Parental food choices and preparation	% of parents who choose low-fat/fat-free foods and beverages for their children (26)
	% of parents who choose low-sodium (salt)/sodium-free foods and beverages for their children (26)
	% of parents who choose low-sugar/sugar-free foods and beverages for their children (26)
	% of parents who choose high-fibre foods and beverages for their children (26)
	% of parents who choose whole grain versions of foods instead of refined grains (26)
	% of parents who choose to use natural herbs and spices and reduce the amount of high-fat/−sodium/−sugar condiments added to foods (e.g., ketchup, barbeque sauce, soy sauces, Nutella) (26)
	% of parents who trim the visible fat or skin off meat before/after cooking foods (26)
	% of parents who rarely cook processed meats (e.g., ham, sausages, bacon, corned beef, and biltong/beef jerky) (26)
	% of parents who rarely cook foods by deep-frying (26)
(4) Home healthier food availability and accessibility	% of parents who make vegetables available in their home (32, 37)
	% of parents who make fruits available in their home (32, 37)
	% of parents who make plain water available in their home (32, 37)
	% of parents who make unhealthy snacks less available in their home (32, 37)
	% of parents who make sugar-sweetened beverages less available in their home (32, 37)
	% of parents who make vegetables accessible for their children (32, 37)
	% of parents who make fruits accessible for their children (32, 37)
	% of parents who make plain water accessible for their children (32, 37)
	% of parents who make unhealthy snacks less accessible for their children (32, 37)
	% of parents who make sugar-sweetened beverages less accessible for their children (32, 37)
(5) Family mealtime environments	% of parents who minimise distractions (i.e., TV, screen devices and toys) during mealtimes (38)
	% of children who eat family meals at a routine time (17)
	% of children who eat the same food as other family members at meals (39)
	% of children who eat home-cooked family meals (31)
	% of children who eat meals at the dining table (rather than eating in the car, etc.) (31)
	% of children who dine with their parents or family members (31)

The predefined benchmarks were used to grade the indicators of the International Healthy Eating Report Card for Preschool-Aged Children. The letter grade for each indicator was determined by calculating the arithmetic mean of the items within the indicator. The benchmarks for each indicator were defined based on the recommendations and guidelines from global health authorities (27–34) and the relevant literature (17, 35–41).

* The five main food groups include (1) grains; (2) fruits; (3) vegetables; (4) meat, fish, eggs, and alternatives; and (5) milk and alternatives.

**Table 3:** Grading system in the International Healthy Eating Report Card for Preschool-Aged Children.

Grade	Percentage and grade explanation	Description
A+	94–100%	
A	Most children/parents meet the predefined benchmark (87–93%)	Excellent
A−	80–86%	
B+	74–79%	
B	More than half of children/parents meet the predefined benchmark (67–73%)	Good
B−	60–66%	
C+	54–59%	
C	Approximately half of children/parents meet the predefined benchmark (47–53%)	Fair
C−	40–46%	
D+	34–39%	
D	Fewer than half of children/parents meet the predefined benchmark (27–33%)	Poor
D−	20–26%	
F	Very few children/parents meet the predefined benchmark (< 20%)	Very Poor

The grading system of the International Healthy Eating Report Card for Preschool-Aged Children was derived from the grading rubric of a Healthy Eating Report Card for Pre-School Children in Hong Kong (23).

All the statistical analyses were conducted using IBM SPSS Statistics 27. The nonresponse rates for the items of the IHERCS were low, ranging from 0 to 0.2%. A descriptive statistical analysis was conducted to determine the percentage of valid answers and 95% confidence intervals (CIs) for each item within the indicators. We then calculated the arithmetic mean for each indicator to determine the grades for the indicators of children’s eating behaviours and FHFEs, as well as the overall report card grade (average grade of all indicators) for each cultural context. The average grades across the four cultural contexts were also calculated. Pearson correlation coefficients were performed to examine the relationships between the scores of IHERCS and sociodemographic characteristics. A one-way analysis of covariance (ANCOVA) was employed to examine the differences in the mean scores of children’s eating behaviours, FHFEs and overall report card between the four cultural contexts. Certain sociodemographic characteristics (e.g., continuous variable: parent’s age and number of children and dichotomous variable: non-working/working mother and below/above median household income) had a significant zero-order correlation with these scores. Therefore, they served as covariates in a general linear model. The normality assumption was assessed using skewness (within the range of −2 to 2) and kurtosis (within the range of −7 to 7) (41). The Levene’s test supported the assumptions for homogeneity of variance for parametric tests (*p* > 0.05). The Bonferroni’s post-hoc test was subsequently performed to determine how the scores of IHERCS differed between the four cultural contexts. A significance level of *p* < 0.05 (two-tailed) was applied in all the tests.

## Results

The average overall report card grade across the four cultural contexts was “B−” (Good), with a range from “C+” (Singapore and the US) to “B−” (Australia and Hong Kong), indicating that, on average, more than half of the children (59.94%; 95% CI [59.44, 60.57]) were engaged in healthy eating practices. The average grade for indicators of Children’s Eating Behaviours for four cultural contexts was “C−” (Fair). Conversely, the average grade for indicators of FHFEs was “B+” (Good) for all cultural contexts. On average, fewer than half of the children (43.67%; 95% CI [42.83, 44.68]) adhered to healthy eating behaviours, while more than half of the children (76.21%; 95% CI [75.75, 76.76%]) were involved in favourable FHFEs. The final grades of the International Healthy Eating Report Card for Preschool-Aged Children across the four cultural contexts are summarised in Table 4. The related descriptive statistics are displayed in Appendix B.

**Table 4:** Letter grades assigned to indicators and their items in the International Healthy Eating Report Card for Preschool-Aged Children among four cultural contexts.

Indicators and their items	Australia	Hong Kong	Singapore	US	Average grade
**Average grade for Indicators of Children’s eating Behaviours**	**C− (Fair)**	**C− (Fair)**	**C− (Fair)**	**C− (Fair)**	**C− (Fair)**
**Indicator: Children’s Dietary Patterns**	**C (Fair)**	**C− (Fair)**	**C− (Fair)**	**C− (Fair)**	**C− (Fair)**
Regular breakfast consumption	A−	A−	B	B+	B+
Not having a formula milk−drinking habit	A	C	C−	A−	B−
Diet diversity within the five main food groups	F	F	F	F	F
Adequate water intake daily	D+	D	D+	C	D+
Adequate vegetable intake daily	D+	C−	D	D	D+
Adequate fruit intake daily	C	D−	D−	C−	D+
Healthy snacks between meals	D−	D	D−	F	D−
Low consumption of unhealthy snacks	C−	C	C	D	C−
Low consumption of sugar−sweetened beverages	B+	B	B	C+	B
**Indicator: Children’s Mealtime Behaviours**	**C− (Fair)**	**C (Fair)**	**C− (Fair)**	**C− (Fair)**	**C− (Fair)**
Willingness to eat a variety of foods (non−picky eaters)	D	C−	D	D	D+
Reasonable eating speed	D−	D	D−	D	D−
Remaining seated at the table during mealtime	D+	C−	D+	D+	D+
Food acceptance (not refusing to eat)	C	B−	C	C	C+
Self−feeding independence	C+	C	D+	B	C
Appropriate portion size consumption	C−	C	C−	D+	C−
Emotion regulation during mealtimes (not throwing a tantrum)	C+	C+	C	C+	C+
**Average grade for Indicators of Family Home Food Environments**	**B+ (Good)**	**B+ (Good)**	**B+ (Good)**	**B+ (Good)**	**B+ (Good)**
**Indicator: Parental Food Choices and Preparation**	**B+ (Good)**	**A− (Excellent)**	**B+ (Good)**	**B+ (Good)**	**B+ (Good)**
Selection of low−fat/fat−free foods and beverages	B−	B	B+	B	B
Selection of low−sodium (salt)/sodium−free foods and beverages	B	A−	A−	B	B+
Selection of low−sugar/sugar−free foods and beverages	B+	A−	A−	B	B+
Selection of high−fibre foods and beverages	A	A	A−	A−	A−
Selection of whole grain versions of foods	A−	A−	B+	B+	B+
Reduction of the amount of high−fat/−sodium/−sugar condiments added to foods	B+	B+	A−	B	B+
Trimming visible fat or skin from meat	B+	B	B+	B+	B+
Avoidance of processed meats in cooking	B+	A−	B+	B	B+
Avoidance of deep−frying cooking methods	A	A	B+	A−	A−
**Indicator: Home Healthier Food Availability and Accessibility**	**B+ (Good)**	**B (Good)**	**B (Good)**	**B (Good)**	**B (Good)**
Home availability of vegetables	A+	A+	A	A+	A+
Home availability of fruit	A+	A	A	A+	A
Home availability of plain water	A+	A+	A	A+	A+
Limited home availability of unhealthy snacks	D−	C−	D	D−	D
Limited home availability of sugar−sweetened beverages	C	D+	D+	D	D+
Home accessibility of vegetables	A−	B	B+	A−	B+
Home accessibility of fruit	A	A−	B+	A	A−
Home accessibility of plain water	A+	A	A	A	A
Limited home accessibility of unhealthy snacks	C	C	C−	D+	C−
Limited home accessibility of sugar−sweetened beverages	B−	C	C	C−	C
**Indicator: Family Mealtime Environments**	**A− (Excellent)**	**A− (Excellent)**	**B+ (Good)**	**A− (Excellent)**	**A− (Excellent)**
Avoidance of distraction during mealtimes (i.e., TV, screen devices and toys) during mealtimes	D−	D−	D	D−	D−
Consistent family mealtime schedule	A+	A	A−	A−	A
Same food as other family members	A+	A	A	A+	A
Frequent home−cooked family meals	A+	A	A	A+	A+
Meal setting at dining table	A	A	A	A−	A
Family mealtime participation	A+	A+	A	A+	A+
**Average grade for Overall Report Card**	**B− (Good)**	**B− (Good)**	**C+ (Fair)**	**C+ (Fair)**	**B− (Good)**

The letter grades for each indicator were determined based on the percentage of children who met the predefined benchmarks for that indicator [i.e., A+ (94–100%), A (87–93%), A− (80–86%); B+ (74–79%), B (67–73%), B− (60–66%); C+ (54–59%), C (47–53%), C− (40–46%); D+ (34–39%), D (27–33%), D− (20–26%); and F (<20%)]. The average grade for indicators of Children’s eating Behaviours = combined indicator grades of Children’s Dietary Patterns and Children’s Mealtime Behaviours. The average grade for indicators of Family Home Food Environment = combined indicator grades of Parental Food Choices and Preparation, Home Healthier Food Availability and Accessibility and Family Mealtime Environments. The average grade for Overall Report Card = combined indicator grades of all indicators.

### Letter grades for indicators of children’s eating behaviours

The average grade for Children’s Dietary Patterns was “C−” (Fair) across the four cultural contexts, ranging from “C−” (Hong Kong, Singapore and the US) to “C” (Australia). The sub-grades of the items ranged from “F” (12.53%; 95% CI [11.13, 14.00]) for diet diversity within the five main food groups to “B+” (77.90%; 95% CI [76.05, 79.65]) for regular breakfast consumption. The average grade for Children’s Mealtime Behaviours was “C−” (Fair) across the four cultural contexts, ranging from “C−” (Australia, Singapore and the US) to “C” (Hong Kong). The sub-grades varied from “D−” (26.42%; 95% CI [24.51, 28.33]) for reasonable eating speed to “C+” (54.98%; 95% CI [52.83, 57.13]) for emotion regulation during mealtimes.

### Letter grades for indicators of FHFEs

The average grade for Parental Food Choices and Preparation was “B+” (Good) across the four cultural contexts, ranging from “B+” (Australia, Singapore and the US) to “A−” (Hong Kong). The sub-grades of the items ranged from “B” (69.31%; 95% CI [67.31, 71.30]) for selection of low-fat/fat-free foods and beverages to “A−” (85.53%; 95% CI [84.01, 87.05]) for selection of high-fibre foods and beverages. The average grade for Home Healthier Food Availability and Accessibility was “B” (Good) across the four cultural contexts, ranging from “B” (Hong Kong, Singapore and the US) to “B+” (Australia). The sub-grades ranged from “D” (29.58%; 95% CI [27.60, 31.55]) for limited home availability of unhealthy snacks to “A+” (95.24%; 95% CI [94.32, 96.16]) for home availability of plain water. The average grade for Family Mealtime Environments was “A−” (Excellent) across the four cultural contexts, ranging from “B+” (Singapore) to “A−” (Australia, Hong Kong and the US). The sub-grades ranged from “D−” (23.31%; 95% CI [21.48, 25.14]) for avoidance of distraction during mealtimes to “A+” (95.00%; 95% CI [94.06, 95.94]) for family mealtime participation.

### Differences in the scores of the report card among the four cultural settings

The correlation coefficients between the scores of the report card and the demographic variables and the means and results of the ANCOVA for the scores of children’s eating behaviours, FHFEs and overall report card among the four cultural contexts are presented in Tables 5, 6, respectively. ANCOVA showed that the score of children’s eating behaviours [*F*(3,2008) = 8.56, *p* < 0.001] was statistically different between the four cultural contexts after controlling for the effects of the dichotomous covariates (i.e., working mothers and two-parent families). The Bonferroni’s *post hoc* analysis revealed that the score of children’s eating behaviours in Australia and Hong Kong was significantly greater than that in Singapore (*p* < 0.001). Moreover, there was a significant difference in the score of FHFEs [*F*(3,1796) = 9.92, *p* < 0.001] between the four cultural contexts after controlling for parent’s age and education level, number of children, child’s age and the dichotomous covariates (i.e., working father, two-parent families, above-median household income). The Bonferroni’s *post hoc* analysis revealed that the score of FHFEs in Australia was significantly greater than that in Hong Kong (*p* = 0.020), Singapore (*p* < 0.001) and the US (*p* = 0.001). For the score of the overall report card, a significant difference [*F*(3,1791) = 11.56, *p* < 0.001] was observed between the four cultural contexts after controlling for parent’s age and dichotomous covariates (i.e., working parents and above median household income). The Bonferroni’s *post hoc* analysis revealed that the overall report card score in Australia was significantly greater than that in Singapore (*p* < 0.001) and the US (*p* = 0.001), while the score in Hong Kong was significantly greater than that in Singapore (*p* < 0.001).

**Table 5:** Correlation matrix.

	1	2	3
1. Score of Children’s Eating Behaviours	−		
2. Score of FHFEs	0.23^**^	−	
3. Score of Overall Report Card	0.81^**^	0.76^**^	−
Continuous control variables			
4. Parent’s age	0.02	0.06^**^	0.05^*^
5. Parent’s level of education	−0.02	0.08^**^	0.03
6. Number of children	0.01	−0.08^**^	−0.04
7. Child’s age	0.02	−0.05^*^	−0.02
Dichotomous control variables			
8. Working fathers	0.02	0.10^**^	0.07^**^
9. Working mothers	−0.08^**^	0.00	−0.05^*^
10. Two-parent families	−0.05^*^	0.09^**^	0.03
11. Above median household income	0.03	0.09^**^	0.08^**^
12. Child’s sex	−0.02	−0.02	−0.03
13. Child attending school	0.03	0.02	0.03

^*^p < 0.05. ^**^p < 0.01.

**Table 6:** Means, standard deviations, and results of analysis of covariance (ANCOVA) for the scores on the report card among the four cultural settings.

Scores	Australia (*n* = 500) Mean (*SD*)	Hong Kong (*n* = 552) Mean (*SD*)	Singapore (*n* = 507) Mean (*SD*)	US (*n* = 500) Mean (*SD*)	Average (*N* = 2059) Mean (*SD*)	*F*	*p*	Partial *η*^2^	*Post hoc* test: significant differences
Children’s eating behaviours	7.35 (3.33)	7.33 (3.29)	6.35 (3.3)	6.98 (3.2)	7.01 (3.3)	8.56	<0.001	0.013	AU versus SG: *p* < 0.001 HK versus SG: *p* < 0.001
Family home food environments	19.39 (2.86)	19.23 (2.85)	18.52 (3.19)	18.36 (3.09)	18.88 (3.03)	9.92	<0.001	0.016	AU versus HK: *p* = 0.020 AU versus SG: *p* < 0.001 AU versus US: *p* = 0.001
Overall report card	26.73 (4.88)	26.56 (4.96)	24.88 (4.89)	25.33 (4.93)	25.89 (4.97)	11.56	<0.001	0.019	AU versus SG: *p* < 0.001 AU versus US: *p* = 0.001 HK versus SG: *p* < 0.001

AU, Australia; HK, Hong Kong; SG, Singapore; US, The United States. The mean difference is significant at the 0.05 level.

## Discussion

This study applied the International Healthy Eating Report Card for Preschool-Aged Children to assess the prevalence of healthy eating behaviours and FHFEs in Australia, Hong Kong, Singapore and the US. We examined the differences in the report card scores between these four cultural contexts. The traditional letter grading system (i.e., A+ to F) was employed to comprehensively assess the prevalence of healthy eating behaviours and favourable FHFEs among young children. The findings indicated that, on average, the eating behaviours of preschool-aged children in these developed Asian and Western societies were classified as Fair (“C−”), representing only fewer than half of the children who could meet the international standards and recommendations for healthy eating behaviours; while FHFEs were, on average, classified as Good (“B+”), representing more than half of the children got involved in the favourable FHFEs among these cultural contexts. Notably, the sub-grades in the indicators of FHFEs exhibited a wide range, extending from “D−” (i.e., avoidance of distraction during mealtimes) to “A+” (e.g., home availability of plain water). Therefore, the elevated sub-grades in certain aspects of FHFEs may increase the average grade of its indicator. These findings also highlighted the need for targeted interventions to enhance children’s eating behaviours and particular aspects of FHFEs.

In summary, all cultural settings achieved the average grade for the overall report card of “B−,” except for Singapore and the US with a grade of “C+.” When comparing the overall report card score, Australia demonstrated a significantly higher overall report card score than Singapore and the US, while Hong Kong also achieved a significantly higher score than Singapore. These findings may suggest that Australia appears to be more successful at promoting healthy eating in young children, whereas Singapore encounters more challenges in upholding the standards of healthy eating behaviours and favourable FHFEs for young children, as compared to the other cultural contexts.

### Cross-cultural comparison of children’s eating behaviours

The grades for indicators of Children’s Dietary Patterns are comparable across Australia, Hong Kong, Singapore, and the US (i.e., C vs. C− vs. C− vs. C−), demonstrating a global trend towards unhealthy dietary patterns among young children. In particular, our findings revealed that a few children (12.53%) consumed all five main food groups daily, so diet diversity within the five main food groups received only a sub-grade of “F” across all cultural contexts examined. Moreover, on average, fewer than half of the children could meet the recommended daily intake of water (38.76%), vegetables (34.66%) or fruit (36.20%) with the sub-grades of of “D+,” respectively. Therefore, this study identified potential areas for improvement, including increasing dietary variety within healthful food groups and ensuring adequate intake of fluids and fibres. These observations are consistent with global concerns about insufficient fruit and vegetable consumption in young children (42). Our findings also revealed that the sub-grades in the US for healthy snacks between meals and low consumption of unhealthy snacks and sugar-sweetened beverages (“F” to “C+”) fell below the average grades (“D−” to “B”). This finding indicates a greater trend towards unhealthy snacking among children in the US. A study reported a large increasing trend in daily unhealthy snacking among children in the US, accounting for up to 27% of the daily caloric intake (43). Therefore, the US government should probably place a high emphasis on implementing intervention policies and programmes to reduce unhealthy snack consumption.

Furthermore, there is a notably higher prevalence of a toddler formula milk-drinking habit among preschool-aged children with a lower sub-grade for not having a formula milk-drinking habit in Asian societies, such as Hong Kong (48.19%; sub-grade of “C”) and Singapore (57.40%; sub-grade of “C−”), compared to Western societies where the prevalence rates are 11.40% (sub-grade of “A”) and 18.00% (sub-grade of “A−”) for children in Australia and the US, respectively. This may be attributed to extensive marketing strategies for toddler formula milk in Asian societies (44). Toddler formula milk often contains high amounts of added sugar, vegetable oil, and sodium while having lower protein contents than cow’s milk, so excessive dependence on formula milk may result in higher sweetness preferences and poorer appetites in children, adversely affecting children’s development of healthy eating patterns (45–47). Therefore, governments should encourage healthier dietary practices from an early age and address the misleading marketing tactics used to promote commercial milk formula, which influence parents’ decisions on infant or toddler feeding (40).

The indicator of Children’s Mealtime Behaviours received similar grades among the four cultural contexts (i.e., C− vs. C vs. C− vs. C−) indicating that, on average, fewer than half of the children (43.16%) had favourable mealtime behaviours. Inappropriate mealtime behaviours, such as picky eating, refusal to self-feed, eating very slowly and throwing tantrums, are prevalent worldwide among typically developing children because it has been estimated that nearly 25–45% of children with typical development experience some form of mealtime behaviour problems (35, 48–51). In line with these global trends, our study showed that inappropriate mealtime behaviours are prevalent among young children. A structured and supportive family meal setting, especially with minimal distractions and established mealtime routines, plays an important role in reducing the likelihood of inappropriate mealtime behaviours in preschool-aged children (e.g., picky eating and slow eating) (18, 52). Consequently, health initiatives should focus on promoting a pleasant and structured home food environment in family settings by educating families on the importance of a favourable mealtime environment for children’s eating behaviours and strategies to support a positive mealtime environment, namely allowing children to make food choices, sharing meals at home and minimising distractions (e.g., TV and screen devices) during mealtimes (18, 53).

### Cross-cultural comparison of FHFEs

Given the limited understanding of FHFEs in different cultural contexts, this study could address this gap by providing novel insight into the extent to which parents could create a healthy home food environment for their children. The indicator of Parental Food Choices and Preparation (i.e., B+ vs. A− vs. B+ vs. B+), Home Healthier Food Availability and Accessibility (i.e., B+ vs. B vs. B vs. B) and Family Mealtime Environments (A− vs. A− vs. B+ vs. A−) were classified as “Good” or “Excellent” among Australia, Hong Kong, Singapore, and the US. These countries/regions are considered developed with a basic level of education and higher standard of living. Therefore, parents in these developed societies may have better knowledge of general health and a higher awareness of healthy eating and preventive measures, leading them to choose prepacked food and prepare meals in healthy ways (54). Moreover, they could have the resources or abilities to create a more favourable home food environment, for example, ensuring that foods are readily available and easily accessible in the home while also providing general mealtime settings to their children where they could dine at the table and consume the same food as their parents or family members (55). The letter grade of the indicators of FHFEs, therefore, tended to be relatively higher. However, there is room for improvement in the specific areas of FHFEs.

First, while most parents in the four cultural contexts (78.00–95.24%) ensured that vegetables, fruits, and water were readily available in their homes and placed in areas easily accessible to children, fewer than half of the parents (29.58–52.21%) could limit the availability and accessibility of unhealthy snacks and sugar-sweetened beverages. A relatively lower average grade, ranging from “C” to “D,” was assigned to the indicator items of limited home availability and accessibility of unhealthy snacks and sugar-sweetened beverages, respectively. Food availability and accessibility in the home are highly associated with one’s dietary consumption, including both healthy (e.g., fruits and vegetables) and unhealthy foods (e.g., junk snacks and sugary drinks) (16, 37, 56–58). Notably, in the US, the lowest percentage of parents could make unhealthy snacks and sugary beverages less available and accessible (20.20–45.00%) within the home, which aligns with the previously highlighted higher prevalence of unhealthy snacking among the US children. Therefore, health initiatives should emphasise reducing the availability and accessibility of unhealthy foods within the home environment in order to lower the consumption of unhealthy snack foods among young children (59). It also underscores the importance of monitoring the effects of favourable FHFEs on children’s healthy eating behaviours providing valuable insights for developing targeted interventions.

Second, in the four cultural contexts, most parents could offer structured family mealtime environments, including regular meal schedules and designated locations for meals and sharing meals with their family members. However, on average, only 23.31% of parents reduced distractions (e.g., screen devices and toys) for their children during mealtimes, with an average sub-grade of “D−.” Our findings align with Goh and Jacob’s (60) findings that most Singaporean parents (89.90%) encouraged their children to eat with their family, but only approximately one-third of them (37.60%) prevented their children from watching TV during mealtime. Importantly, children who are not prohibited from watching screen devices o during mealtimes have been shown to be more likely to consume unhealthy foods and to exhibit a higher prevalence of behavioural problems during mealtimes (61). Therefore, our findings emphasised the importance of reducing distractions during mealtimes to promote a more favourable mealtime environment for young children. Nutrition education programmes for parents are crucial for promoting healthy food choices, preparation and supportive home food environments. For instance, Fulkerson et al. (62) developed the Healthy Home Offerings via the Mealtime Environment (HOME) Plus Programme to provide nutrition education and activities for parents and children, enhancing parents’ nutrition knowledge, facilitating healthier home food environments and ultimately optimizing children’s health. Future healthy eating programmes should focus on enhancing nutrition education for parents to underscore the importance of favourable FHFEs in promoting children’s healthy eating behaviours.

### Strengths and limitations

There are several strengths in our present study. First, we employed a systematic and comprehensive framework for assessing children’s eating behaviours and FHFEs in four cultural contexts, including Australia, Hong Kong, Singapore and the US. To the authors’ knowledge, this is the first study to adopt the International Healthy Eating Report Card for Preschool-Aged Children to assess and compare the status of healthy eating among these geographical regions. This approach not only enhances the comparability of data across diverse cultural contexts, but it also may ensure consistency in the interpretation of results. Moreover, we measured children’s eating behaviours and FHFES using the cross-culturally validated questionnaire (i.e., IHERCS). It was tailored to assess the indicators of the International Healthy Eating Report Card for Preschool-Aged Children to ensure reliable measurement and valid results (24). As such, our study could provide a robust model for future research, fostering international collaboration and comparative studies to further enhance our understanding of global eating behaviours and FHFEs among preschool-aged children.

Despite the strengths of our study, certain limitations should be mentioned. A major limitation is that we relied solely on the parent-reported measure to assess children’s eating behaviours and FHFEs, which may be susceptible to several response biases, including social desirability, recall error, acquiescence, and extreme responses (63–65). To mitigate these biases, we implemented consistency checks through an online survey panel to identify implausible or impossible answers (e.g., the combination of parent’s age and their children’s age) for the detection and exclusion of low-quality datasets (66). A unique IP address check was employed to minimise the potential biasing effect of multiple submissions. Future studies could also enhance methodological rigour by integrating objective measures or observational techniques (e.g., dietary recalls). Another limitation is the adoption of a cross-sectional study design in this study. Although such a design made it easier to obtain international data, which offers valuable insights into cross-cultural comparisons, it limits the ability to reveal changes or the stability of children’s healthy eating patterns over time. Future research should consider adopting a longitudinal design to examine the temporal stability and predictive power of the scores of the report card. It is also important that future studies include other objective outcome measures, namely a child’s BMI, fat percentage, muscle mass, and functional fitness, so we can understand if the report card scores are predictive of young children’s health status over time (67). Finally, this study focused primarily on assessing the overall prevalence of healthy eating within regions or countries, but we did not take the variations in the cultural and ethnic compositions of the collected samples into account. Future research could address these limitations by providing a comprehensive evaluation of the cultural and ethnic differences in healthy eating behaviours and FHFEs within a geographical region.

## Conclusion

The present study initially adopted the International Healthy Eating Report Card for Preschool-Aged Children to assess the extent to which children in four cultural settings (i.e., Australia, Hong Kong, Singapore and the US) adhere to the standards and recommendations of healthy eating patterns and favourable FHFEs. Our findings showed that, on average, Children’s Eating Behaviours were classified as Fair (“C−”), while FHFEs were classified as Good (“B+”). The study also highlighted the need for improvements in diet diversity within the five main food groups, as well as in the consumption of vegetables, fruits, and water to promote healthy dietary patterns in children. In addition, reducing the availability and accessibility of unhealthy foods in the home environment, along with minimising distractions at mealtime, are shown to be pivotal factors in fostering a healthy home food environment. Future interventions can specifically target these areas to improve children’s healthy eating optimising their health. When comparing the differences in the report card scores between the four cultural contexts, the results suggested that Australia and Singapore appear to be the most and the least successful regions, respectively, in promoting healthy eating among young children. The lower scores observed in Singapore suggested that local health organizations should increase their efforts to support and enhance healthy eating practices among young children. We believe that the International Healthy Eating Report Card may offer new perspectives on interventions promoting healthy eating behaviours and FHFEs. It serves as a novel tool for providing a comprehensive assessment of the prevalence of healthy eating and advocating for supportive policies or programmes to promote healthy dietary habits in preschool-aged children.

## Data availability statement

The data analysed in this study is subject to the following licenses/restrictions: the raw data supporting the conclusions of this article will be made available by the authors, without undue reservation. Requests to access these datasets should be directed to derwin@eduhk.hk.

## Ethics statement

The studies involving humans were approved by the Human Research Ethics Committee of the Education University of Hong Kong. The studies were conducted in accordance with the local legislation and institutional requirements. The participants provided their written informed consent to participate in this study.

## Author contributions

AW: Conceptualization, Data curation, Formal analysis, Investigation, Methodology, Project administration, Validation, Writing – original draft, Writing – review & editing. KC: Conceptualization, Investigation, Project administration, Supervision, Writing – review & editing. J-BL: Conceptualization, Investigation, Project administration, Supervision, Writing – review & editing. SX: Formal analysis, Investigation, Validation, Writing – review & editing, Methodology. DC: Conceptualization, Funding acquisition, Investigation, Project administration, Resources, Supervision, Validation, Writing – review & editing.
