# Supplementary material for: A report card assessment of the prevalence of healthy eating among preschool-aged children: a cross-cultural study across Australia, Hong Kong, Singapore, and the US
Source: Front Nutr. 2024 Aug 21;11:1428852. doi: 10.3389/fnut.2024.1428852 (PMC11371745; doi:10.3389/fnut.2024.1428852)
Supplement: Supplementary file 1 [file Table_1.docx]

Appendix A. Corresponding IHERCS Items and Response Criteria for Meeting Predefined Benchmarks

| **Benchmark** | **Corresponding item of the IHERCS** | **Question type** | **Response criteria for meeting benchmark** |
| --- | --- | --- | --- |
| **Indicator: Children’s Dietary Patterns** | | | |
| % of children who eat breakfast daily | Q1.6 | Open-ended question | A respondent answered 7 days. |
| % of children who do not have a formula milk-drinking habit | Q1.7 | Open-ended question | A respondent answered less than three times a week. |
| % of children who eat a variety of foods from each of the five main food groups daily | Q1.1 | Open-ended question | A respondent answered 7 days for each food group. |
| % of children who have an adequate fluid intake daily | Q1.8 | Open-ended question | A respondent answered ≥5.5 glasses/day for children aged 2-3 years old and ≥6.5 glasses/day for children aged 4-8 years old. |
| % of children who have an adequate vegetable intake daily | Q1.2 | Open-ended question | A respondent answered ≥1 cup/day for children aged 2-3 years old or ≥1.5 cup/day for children aged 4-8-year-old. |
| % of children who have an adequate fruit intake daily | Q1.3 | Open-ended question | A respondent answered ≥1 cup/day for children aged 2-3 years old or ≥1.5 cup/day for children aged 4-8 years old. |
| % of children who eat healthy snacks between meals | Q1.4 | Multiple choices (i.e., responding to a list of snack and drink items) | A respondent only selects the healthy foods (asterisk items), indicating that a child has a low consumption of unhealthy snacks that are eaten between meals. |
| % of children who consume unhealthy snacks fewer than three times per week | Q1.5(a-e) | 5-point Likert scale (ranging from Never to Always) | A respondent answered less than three times a week for the items (a-e). |
| % of children who drink sugar-sweetened beverages fewer than three times per week | Q1.5(f) | 5-point Likert scale (ranging from Never to Always) | A respondent answered less than three times a week for the item (f). |
| **Indicator:** **Children’s Mealtime behaviours** | | | |
| % of children who are not picky eaters | Q 2.1 | 5-point Likert scale (ranging from Never to Always) | A respondent answered “Never” or “Rarely” for the item. |
| % of children who are not slow eaters | Q 2.2 |  | A respondent answered “Never” or “Rarely” for the item. |
| % of children who remain seated at the table for most of the meal | Q 2.3 |  | A respondent answered “Never” or “Rarely” for the item. |
| % of children who do not refuse to eat at meals | Q 2.4 |  | A respondent answered “Never” or “Rarely” for the item. |
| % of children who do not require parental feeding assistance to finish most meals | Q 2.5 |  | A respondent answered “Never” or “Rarely” for the item. |
| % of children who eat an adequately sized meal | Q 2.6 |  | A respondent answered “Never” or “Rarely” for the item. |
| % of children who do not throw a tantrum during mealtimes | Q 2.7 |  | A respondent answered “Never” or “Rarely” for the item. |

| **Indicator: Parental Food Choices and Preparation** | | | |
| --- | --- | --- | --- |
| % of parents who choose low-fat/fat-free foods and beverages for their children | Q3.1 | 5-point Likert scale (ranging from Never to Always) | A respondent answered “Sometimes”, “Often” or “Always” for the item. |
| % of parents who choose low-sodium (salt)/sodium-free foods and beverages for their children | Q3.2 |  | A respondent answered “Sometimes”, “Often” or “Always” for the item. |
| % of parents who choose low-sugar/sugar-free foods and beverages for their children | Q3.4 |  | A respondent answered “Sometimes”, “Often” or “Always” for the item. |
| % of parents who choose high-fibre foods and beverages for their children | Q3.6 |  | A respondent answered “Sometimes”, “Often” or “Always” for the item. |
| % of parents who choose whole grain versions of foods instead of refined grains | Q3.7 |  | A respondent answered “Sometimes”, “Often” or “Always” for the item. |
| % of parents who choose to use natural herbs and spices and reduce the amount of high-fat/-sodium/-sugar condiments added to foods (e.g., ketchup, barbeque sauce, soy sauces, Nutella) | Q3.9 |  | A respondent answered “Sometimes”, “Often” or “Always” for the item. |
| % of parents who trim the visible fat or skin off meat before/after cooking foods | Q3.3 |  | A respondent answered “Sometimes”, “Often” or “Always” for the item. |
| % of parents who rarely cook processed meats (e.g., ham, sausages, bacon, corned beef, and biltong/beef jerky) | Q3.5 |  | A respondent answered “Sometimes”, “Often” or “Always” for the item. |
| % of parents who rarely cook foods by deep-frying | Q3.8 |  | A respondent answered “Sometimes”, “Often” or “Always” for the item. |
| **Indicator: Home Healthier Food Availability and Accessibility** | | | |
| % of parents who make vegetables available in their home | Q 4.1.a | 5-point Likert scale (ranging from Never to Always) | A respondent answered “Sometimes”, “Often” or “Always” for the item. |
| % of parents who make fruits available in their home | Q 4.2.a |  | A respondent answered “Sometimes”, “Often” or “Always” for the item. |
| % of parents who make plain water available in their home | Q 4.3.a |  | A respondent answered “Sometimes”, “Often” or “Always” for the item. |
| % of parents who make unhealthy snacks less available in their home | Q 4.4.a |  | A respondent answered “Never” or “Rarely” for the item. |
| % of parents who make sugar-sweetened beverages less available in their home | Q 4.5.a |  | A respondent answered “Never” or “Rarely” for the item. |
| % of parents who make vegetables accessible for their children | Q 4.1.b |  | A respondent answered “Sometimes”, “Often” or “Always” for the item. |
| % of parents who make fruits accessible for their children | Q 4.2.b |  | A respondent answered “Sometimes”, “Often” or “Always” for the item. |
| % of parents who make plain water accessible for their children | Q 4.3.b |  | A respondent answered “Sometimes”, “Often” or “Always” for the item. |
| % of parents who make unhealthy snacks less accessible for their children | Q 4.4.b |  | A respondent answered “Never” or “Rarely” for the item. |
| % of parents who make sugar-sweetened beverages less accessible for their children | Q 4.5.b |  | A respondent answered “Never” or “Rarely” for the item. |
| **Indicator: Family Mealtime Environments** | | | |
| % of parents who minimise distractions (i.e., TV, screen devices and toys) during mealtimes | Q 5.1,5.3,5.6 | 5-point Likert scale (ranging from Never to Always) | A respondent answered “Never” or “Rarely” for all the items. |
| % of children who eat family meals at a routine time | Q5.2 |  | A respondent answered “Sometimes”, “Often” or “Always” for the item. |
| % of children who eat the same food as other family members at meals | Q5.4 |  | A respondent answered “Sometimes”, “Often” or “Always” for the item. |
| % of children who eat home-cooked family meals | Q5.5 |  | A respondent answered “Sometimes”, “Often” or “Always” for the item. |
| % of children who eat meals at the dining table (rather than eating in the car, etc.) | Q5.7 |  | A respondent answered “Sometimes”, “Often” or “Always” for the item. |
| % of children who dine with their parents or family members | Q5.8 |  | A respondent answered “Sometimes”, “Often” or “Always” for the item. |

Appendix B. Descriptive Statistics of the Items of the Indicators

| **Items of the Indicator** | **Australia**  **(*n*=500)** | | **Hong Kong**  **(*n*=552)** | | **Singapore**  **(**n**=507)** | | **The United States (*n*=500)** | | **Whole Cultural Settings**  **(*N*=2059)** | |
| --- | --- | --- | --- | --- | --- | --- | --- | --- | --- | --- |
|  | **Freque-ncy** | **Percentage of Meeting the Benchmark**  **[95% CI]** | **Freque-ncy** | **Percentage of Meeting the Benchmark**  **[95% CI]** | **Freque-ncy** | **Percentage of Meeting the Benchmark**  **[95% CI]** | **Freque-ncy** | **Percentage of Meeting the Benchmark**  **[95% CI]** | **Freque-ncy** | **Percentage of Meeting the Benchmark**  **[95% CI]** |
| **Children’s Eating Behaviours** | Mean percentage = 45.36% [43.47-47.26] | | Mean percentage = 46.05% [44.25-47.82] | | Mean percentage = 39.53% [37.8-41.55] | | Mean percentage = 43.71% [41.9-45.55] | | Mean percentage = 43.67% [42.83-44.68] | |
| **Indicator: Children’s dietary Patterns** | Mean percentage = 49.76% [47.91-51.60] | | Mean percentage = 44.23% [42.54-45.99] | | Mean percentage = 39.87% [38.14-41.71] | | Mean percentage = 42.83% [41.06-44.58] | | Mean percentage = 44.17% [43.29-45.08] | |
| Regular breakfast consumption | 404 | 80.80%  [77.34-84.26] | 461 | 83.51%  [80.41-86.62] | 360 | 71.01%  [67.04-74.97] | 379 | 75.80%  [72.03-79.57] | 1604 | 77.90%  [76.05-79.65] |
| Not having a formula milk-drinking habit | 443 | 88.60%  [85.80-91.40] | 286 | 51.81%  [47.63-55.99] | 216 | 42.60%  [38.28-46.92] | 410 | 82.00%  [78.62-85.38] | 1355 | 65.81%  [63.87-67.97] |
| Diet diversity within the five main food groups | 77 | 15.40%  [12.23-18.57] | 85 | 15.40%  [12-38-18.42] | 50 | 9.86%  [7.26-12.47] | 46 | 9.20%  [6.66-11.74] | 258 | 12.53%  [11.13-14.00] |
| Adequate water intake daily | 175 | 35.00%  [30.80-39.20] | 172 | 31.16%  [27.33-35.10] | 197 | 38.86%  [34.81-43.36] | 254 | 50.80%  [46.30-55.10] | 798 | 38.76%  [36.69-40.91] |
| Adequate vegetable intake daily | 175 | 35.00%  [30.80-39.20] | 223 | 40.47%  [36.36-44.58] | 162 | 32.14%  [28.05-36.23] | 152 | 30.50%  [26.41-34.51] | 712 | 34.66%  [32.60-36.72] |
| Adequate fruit intake daily | 267 | 53.40%  [49.01-57.79] | 145 | 26.27%  [22.63-30] | 128 | 25.30%  [21.58-29.21] | 205 | 41.00%  [36.55-45.21] | 745 | 36.20%  [34.14-38.30] |
| Healthy snacks between meals | 112 | 22.40%  [18.73-26.07] | 146 | 26.45%  [22.76-30.14] | 108 | 21.30%  [17.73-24.88] | 52 | 10.40%  [7.72-13.08] | 418 | 20.3%  [18.56-22.04] |
| Low consumption of unhealthy snacks | 208 | 41.60%  [37.26-45.94] | 284 | 51.45%  [47.27-55.63] | 250 | 49.31%  [44.94-53.68] | 158 | 31.6%  [27.51-35.69] | 900 | 43.71%  [41.62-45.95] |
| Low consumption of sugar-sweetened beverages | 378 | 75.60%  [71.82-79.38] | 395 | 71.56%  [67.78-75.33] | 347 | 68.44%  [64.38-72.5] | 271 | 54.20%  [49.82-58.58] | 1391 | 67.56%  [65.55-69.6] |
| **Indicator: Children’s Mealtime Behaviours** | Mean percentage = 40.97% [38.19-43.75] | | Mean percentage = 47.88% [45.16-50.46] | | Mean percentage = 39.19% [36.69-42.16] | | Mean percentage =  44.6% [41.92-47.35] | | Mean percentage = 43.16% [41.95-44.68] | |
| Willingness to eat a variety of foods (non-picky eaters) | 139 | 27.80%  [23.86-31.74] | 243 | 44.02%  [39.87-48.18] | 163 | 32.15%  [28.07-36.23] | 151 | 30.20%  [26.16-34.24] | 696 | 33.80%  [31.76-35.85] |
| Reasonable eating speed | 112 | 22.40%  [18.73-26.07] | 165 | 29.89%  [26.06-33.72] | 128 | 25.25%  [21.45-29.04] | 139 | 27.80%  [23.86-37.74] | 544 | 26.42%  [24.51-28.33] |
| Remaining seated at the table during mealtime | 183 | 36.60%  [32.36-40.84] | 229 | 41.49%  [37.36-45.61] | 192 | 37.87%  [33.63-42.11] | 191 | 38.20%  [33.93-42.47] | 795 | 38.61%  [36.51-40.72] |
| Food Acceptance (not refuse to eat) | 251 | 50.20%  [45.80-54.60] | 347 | 62.86%  [58.82-66.91] | 255 | 50.30%  [45.93-54.66] | 265 | 53.00%  [48.61-57.39] | 1118 | 54.30%  [52.14-56.45] |
| Self-feeding independence | 269 | 53.80%  [49.42-58.18] | 277 | 50.18%  [46.00-54.37] | 181 | 35.70%  [31.52-39.88] | 337 | 67.4%  [63.28-71.52] | 1064 | 51.68%  [49.52-53.84] |
| Appropriate portion size consumption | 206 | 41.20%  [36.87-45.53] | 286 | 51.81%  [47.63-55.99] | 203 | 40.04%  [35.76-44.32] | 192 | 38.40%  [34.12-42.68] | 887 | 43.08%  [40.94-45.22] |
| Emotion regulation during mealtimes (not throwing a tantrum) | 274 | 54.80%  [50.42-59.18] | 303 | 54.89%  [50.73-59.06] | 269 | 53.06%  [48.70-57.42] | 286 | 57.20%  [52.85-61.55] | 1132 | 54.98%  [52.83-57.13] |
| **FHFES** | Mean percentage =  78.16% [77.21-79.12] | | Mean percentage =  77.6% [76.69-78.52] | | Mean percentage = 74.78% [73.7-75.93] | | Mean percentage = 74.32% [73.25-75.37] | | Mean percentage = 76.21% [75.75-76.76] | |
| **Indicator: Parental Food Choices and Preparation** | Mean percentage = 78.24% [76.48-80] | | Mean percentage = 80.66% [79.2-82.12] | | Mean percentage =  78.3% [76.76-79.94] | | Mean percentage = 74.47% [72.60-76.28] | | Mean percentage = 77.92% [77.16-78.83] | |
| Selection of low-fat/fat-free foods and beverages | 323 | 64.60%  [60.39-68.81] | 385 | 69.75%  [65.90-73.59] | 382 | 75.35%  [71.58-79.11] | 337 | 67.40%  [63.28-71.52] | 1427 | 69.31%  [67.31-71.30] |
| Selection of low-sodium (salt)/sodium-free foods and beverages | 362 | 72.40%  [68.47-76.33] | 464 | 84.06%  [80.99-87.12] | 404 | 79.68%  [76.17-83.2] | 346 | 69.20%  [65.14-73.26] | 1576 | 76.54%  [74.71-78.37] |
| Selection of low-sugar/sugar-free foods and beverages | 395 | 79.00%  [75.42-82.58] | 448 | 81.16%  [77.89-84.43] | 412 | 81.26%  [77.85-84.67] | 365 | 73.00%  [69.10-76.90] | 1620 | 78.68%  [76.91-80.45] |
| Selection of high-fibre foods and beverages | 446 | 89.20%  [86.47-91.93] | 491 | 88.95%  [86.33-91.57] | 424 | 83.63%  [80.4-86.86] | 400 | 80.00%  [76.48-83.52] | 1761 | 85.53%  [84.01-87.05] |
| Selection of whole grain versions of foods | 400 | 80.00%  [76.48-83.52] | 441 | 79.89%  [76.54-83.25] | 383 | 75.54%  [71.79-79.30] | 382 | 76.40%  [72.67-80.13] | 1606 | 78.00%  [76.21-79.79] |
| Reduction of the amount of high-fat/-sodium/-sugar condiments added to foods | 388 | 77.60%  [73.93-81.27] | 416 | 75.36%  [71.76-78.97] | 414 | 81.66%  [78.28-85.04] | 357 | 71.40%  [67.43-75.37] | 1575 | 76.49%  [74.66-78.33] |
| Trimming visible fat or skin from meat | 373 | 74.60%  [70.77-78.43] | 396 | 71.74%  [67.97-75.57] | 376 | 74.16%  [70.34-77.99] | 392 | 78.40%  [74.78-82.02] | 1537 | 74.65%  [72.77-76.53] |
| Avoidance of processed meats in cooking | 396 | 79.20%  [75.63-82.77] | 468 | 84.78%  [81.78-87.79] | 387 | 76.33%  [72.62-80.04] | 354 | 70.80%  [66.80-74.80] | 1605 | 77.95%  [76.16-79.74] |
| Avoidance of deep-frying cooking methods | 438 | 87.60%  [84.70-90.50] | 498 | 90.22%  [87.73-92.70] | 391 | 77.12%  [73.45-80.79] | 418 | 83.60%  [80.34-86.86] | 1745 | 84.75%  [83.20-86.30] |
| **Indicator: Home Healthier Food Availability and Accessibility** | Mean percentage = 74.24% [72.86-75.62] | | Mean percentage = 71.12% [69.64-72.5] | | Mean percentage = 67.75% [66.26-69.29] | | Mean percentage = 68.68% [67.32-70.08] | | Mean percentage = 70.45% [69.74-71.18] | |
| Home availability of vegetables | 489 | 97.80%  [96.51-99.09] | 523 | 94.75%  [92.88-96.61] | 461 | 90.93%  [88.42-93.44] | 472 | 94.40%  [92.38-96.42] | 1945 | 94.46%  [93.47-95.45] |
| Home availability of fruit | 479 | 95.80%  [94.04-97.56] | 516 | 93.48%  [91.41-95.54] | 451 | 88.95%  [86.22-91.69] | 477 | 95.40%  [93.56-97.24] | 1923 | 93.39%  [92.32-94.47] |
| Home availability of plain water | 486 | 97.20%  [95.75-98.65] | 526 | 95.29%  [93.52-97.06] | 472 | 93.10%  [90.88-95.31] | 477 | 95.40%  [93.56-97.24] | 1961 | 95.24%  [94.32-96.16] |
| Limited home availability of unhealthy snacks | 106 | 21.20%  [17.61-24.79] | 237 | 42.93%  [38.79-47.08] | 165 | 32.54%  [28.45-36.64] | 101 | 20.20%  [16.67-23.73] | 609 | 29.58%  [27.6-31.55] |
| Limited home availability of sugar-sweetened beverages | 231 | 46.20%  [41.82-50.58] | 202 | 36.59%  [32.56-40.63] | 180 | 35.50%  [31.32-39.68] | 150 | 30.00%  [25.97-34.03] | 763 | 37.06%  [34.97-39.14] |
| Home accessibility of vegetables | 427 | 85.40%  [82.29-88.51] | 377 | 68.30%  [64.40-72.19] | 381 | 75.15%  [71.37-78.92] | 421 | 84.20%  [80.99-87-41] | 1606 | 78.00%  [76.21-79.79] |
| Home accessibility of fruit | 460 | 92.00%  [89.61-94.39] | 454 | 82.25%  [79.05-85.44] | 401 | 79.09%  [75.54-82.64] | 455 | 91.00%  [88.48-93.52] | 1770 | 85.96%  [84.46-87.47] |
| Home accessibility of plain water | 478 | 95.60%  [93.8-97.4] | 514 | 93.12%  [91.00-95.23] | 462 | 91.10%  [88.64-93.61] | 465 | 93.00%  [90.76-95.24] | 1919 | 93.20%  [92.11-94.29] |
| Limited home accessibility of unhealthy snacks | 241 | 48.20%  [43.81-52.59] | 290 | 52.54%  [48.36-56.71] | 214 | 42.21%  [37.9-46.52] | 191 | 38.20%  [33.93-42.47] | 936 | 45.46%  [43.31-47.61] |
| Limited home accessibility of sugar-sweetened beverages | 315 | 63.00%  [58.75-67.25] | 287 | 51.99%  [47.81-56.17] | 248 | 48.92%  [44.55-53.28] | 225 | 45.00%  [40.62-49.38] | 1075 | 52.21%  [50.05-54.37] |
| **Indicator: Family Mealtime Environments** | Mean percentage =  82.00% [80.85-83.15] | | Mean percentage = 81.01% [79.87-82.32] | | Mean percentage = 78.27% [76.65-79.96] | | Mean percentage =  79.80% [78.43-81.15] | | Mean percentage = 80.27% [79.63-80.99] | |
| Avoidance of distraction during mealtimes (i.e., TV, screen devices and toys) during mealtimes | 102 | 20.40%  [16.86-23.94] | 112 | 20.29%  [16.92-23.66] | 145 | 28.60%  [24.65-32.55] | 121 | 24.20%  [20.43-27.97] | 480 | 23.31%  [21.48-25.14] |
| Consistent family mealtime schedule | 468 | 93.60%  [91.45-95.75] | 510 | 92.39%  [90.17-94.61] | 438 | 86.39%  [83.4-89.39] | 417 | 83.40%  [80.13-86.67] | 1833 | 89.02%  [87.67-90.38] |
| Same food as other family members | 471 | 94.20%  [92.14-96.26] | 511 | 92.57%  [90.38-94.77] | 439 | 86.59%  [83.61-89.56] | 469 | 93.80%  [91.68-95.92] | 1890 | 91.79%  [90.61-92.98] |
| Frequent home-cooked family meals | 484 | 96.80%  [95.25-98.35] | 516 | 93.48%  [91.41-95.54] | 451 | 88.95%  [86.22-91.69] | 480 | 96.00%  [94.28-97.72] | 1931 | 93.78%  [92.74-94.83] |
| Meal setting at dining table | 450 | 90.00%  [87.36-92.64] | 508 | 92.03%  [89.76-94.30] | 443 | 87.38%  [84.48-90.28] | 427 | 85.40%  [82.29-88.51] | 1828 | 88.78%  [87.42-90.15] |
| Family mealtime participation | 485 | 97.00%  [95.50-98.50] | 526 | 95.29%  [93.52-97.06] | 465 | 91.72%  [89.31-94.12] | 480 | 96.00%  [94.28-97.72] | 1956 | 95.00%  [94.06-95.94] |
| **Overall Report Card** | Mean Percentage = 61.76% [60.63-62.90] | | Mean Percentage = 61.83% [60.72-62.92] | | Mean Percentage = 57.15% [56.13-58.36] | | Mean Percentage = 59.02% [57.89-60.14] | | Mean Percentage = 59.94% [59.44-60.57] | |
